# Optimized MALDI-TOF MS Strategy for Characterizing Polymers

**DOI:** 10.3389/fchem.2021.698297

**Published:** 2021-06-24

**Authors:** Zhenxin Wang, Quanqing Zhang, Huali Shen, Pengyuan Yang, Xinwen Zhou

**Affiliations:** ^1^ Institutes of Biomedical Sciences of Shanghai Medical School and Laboratory Medicine of Zhongshan Hospital, Fudan University, Shanghai, China; ^2^ Department of Chemistry, University of California, Riverside, CA, United States; ^3^ Institutes of Biomedical Sciences of Shanghai Medical School and Minhang Hospital, Fudan University, Shanghai, China; ^4^ Institutes of Biomedical Sciences of Shanghai Medical School and Department of Chemistry, Fudan University, Shanghai, China; ^5^ Institutes of Biomedical Sciences of Shanghai Medical School, Fudan University, Shanghai, China

**Keywords:** MALDI-TOF MS, polymer, polyvinylpyrrolidone K12, polymer polyol KPOP-5040, polyether polyol DL-4000, polyethylene glycol 6000

## Abstract

In recent years, matrix-assisted laser desorption/ionization time-of-flight mass spectrometry (MALDI-TOF MS) plays an essential role in the analysis of polymers. To acquire a more reliable strategy for polymer profiling, we characterized four representative polymers including polyethylene glycol 6000, polyvinylpyrrolidone K12, polymer polyol KPOP-5040, and polyether polyol DL-4000. The preparation methods of these four polymer samples have been optimized from six aspects, including matrix, cationization reagent, solvent, mixing ratio of cationization reagent to polymer, mixing ratio of matrix to polymer, and laser intensity. After investigating the effects of seven commonly used matrices on the ionization efficiency of four polymers, *trans*-2-[3-(4-tert-butylphenyl)-2-methyl-2-propenylidene] malononitrile (DCTB) was found to be the only matrix suitable for the analysis of all the four polymers. Our experimental results suggested that different polymers showed a certain preference for different cationization reagents. For example, the polymer polyol KPOP-5040 was suitable for sodium iodide as the cationization reagent, while polyvinylpyrrolidone K12 was more suitable for silver trifluoroacetate (AgTFA). For the choice of solvent, tetrahydrofuran is a reagent with rapid evaporation and a wide range of dissolution which can achieve the best results for the analysis of four polymers. The optimized method was successfully applied to the identification of DSPE-PEG-NH_2_ with different polymerized degrees. This MALDI-TOF strategy potentially provided the supplementary function through the polymer’s application in biomedical and visible probing.

## Introduction

Compared with electrospray mass spectrometry (MS), matrix-assisted laser desorption/ionization time-of-flight mass spectrometry (MALDI-TOF MS) has the characteristics of high sensitivity, easy operation, and wide application (Danis et al., 1992; Roy et al., 1995; Nielen, 1999; Falkenhagen and Weidner, 2010; Peng and Kinsel, 2010). With the development of MALDI-TOF MS, it is increasingly used in the research of polymer structure analysis, including the number of repeating polymer units, molecular weight distribution, terminal structure, and other information, which could perfectly complement the other traditional techniques such as NMR and XRD. Sample preparation is one of the most critical steps for MALDI-TOF MS (Michael et al., 1985; Zhang and Kinsel, 2002) analysis. Since polymers have the characteristics of large molecular weight range and limited solubility, optimization for the polymer preparation method is significant (Hanton and Parees, 2005).

The molecular weight ranges from several hundreds to a few millions in different polymers. In the meantime, the structure of polymers is also complex and diverse. Therefore, a suitable polymer sample solvent, matrix, cationization reagent, instrument parameter, and spotting method, etc., are the key conditions for the sample preparation method of MS-based polymer characterization (Juhasz et al., 1993; Dai et al., 1996; Bauer et al., 2010; Skelton et al., 2013). Six aspects need to be considered when performing polymer MALDI-TOF MS analysis, including the compatibility of different polymers and matrices, the compatibility of different polymers and cationization reagent solvents, with the best selection of matrices and cationization reagents, taking into account the proportional relation between different polymers and the matrix, the proportional relation between different polymers and the cationization agent, the volatilization of mixed solvents, and the uniform cocrystallization. In this study, MALDI-TOF MS was used to characterize four representative polymers including polyethylene glycol 6000(PEG-6000), polyvinylpyrrolidone K12 (PVPK12), polymer polyol KPOP-5040 (KPOP-5040), and polyether polyol DL-4000 (DL-4000). At the same time, the preparation methods of these four polymer samples had also been further optimized in this study.

To determine the molecular weight of polymers by MS is important to illustrate their structure and function. PEG is widely used in industrial and consumer products. During biochemical assays, PEG is always employed to partition proteins in aqueous two-phase systems (Singh and Tavana, 2018). Recently, PEG was described as a high-risk hidden allergen in drug and food items that can induce allergic reactions and diseases (Przepiorka et al., 1980). A research suggested that PEG may have a potential role in the allergic reactions to the COVID-19 vaccine, and for patients testing positive, avoidance of PEG and PEG analogues is strictly recommended (Cabanillas et al., 2020). PVP is widely used in automobile, furniture, and petroleum industries and as the main raw materials of curing waterproof coating; furthermore, it was also found to be better formulations to achieve introducing a clinically relevant hydrophobic anticancer drug into self-assembled nanoparticles successfully (Chowdhury et al., 2018; Schupp et al., 2018). The use of the polyether polyol enables the production of a low-density, low-hardness flexible urethane foam using a small amount of water, without requiring any environment-unfriendly chlorofluorocarbon and without being accompanied by deterioration of humid age compression set and other characteristics. Therefore, it is essential to generate a widespread method for polymer detection. We took a comparative work on the selection of matrix, solvent, and different proportions of polymer detection conditions, aiming at the promotion of polymer ionization efficiency and mass spectrum signal.

## Experiment

### Reagents and Materials

PEG-6000 and PVPK12 were purchased from Sigma-Aldrich (St. Louis, MO, United States). KPOP-5040 was purchased from Guodu Chemical Company (Kunshan, Jiangsu, China). DL-4000 was purchased from Bluestar Dongda Chemical Company (Zibo, Shandong, China). 2,5-Dihydroxybenzoic acid (DHB), *trans*-2-[3-(4-tert-butylphenyl)-2-methyl-2-propenylidene] malononitrile (DCTB), dithranol, 2,3,4-trihydroxyacetophenone (2,3,4-THAP), 2,4,6-trihydroxyacetophenone (2,4,6-THAP), a-cyano-4-hydroxycinnamic acid (CHCA), 9-aminoacridines (9AA), silver trifluoroacetate (AgTFA),sodium iodide, and sodium trifluoroacetate (NaFTA) were purchased from Sigma-Aldrich (St. Louis, MO, United States). High-performance liquid chromatography (HPLC)-grade chloroform, acetonitrile, tetrahydrofuran, methanol, acetone, and trifluoroacetic acid were purchased from Merck Millipore (Billerica, MA, United States). The water used in all experiments was prepared in a Milli-Q water purification system and displayed a resistivity of ≥18.2 MΩ cm^−1^.

### Instruments

The centrifugal dryer (Concentrator plus), high-speed centrifuge (Centrifuge, 5417R), and thermomixer comfort were produced by Eppendorf (Germany). The BS110S precision balance was produced by Sartorius (Germany). The MALDI-T0F/TOF 5800 system was produced by SCIEX (United States).

### Experimental Procedure

#### Effects of Matrix

Seven commonly used matrices (CHCA, DHB, DCTB, dithranol, 9AA, 2,3,4-THAP, and 2,4,6-THAP) were weighted separately and dissolved in tetrahydrofuran to prepare a 20 mg/ml solution. Three cationization reagents (silver trifluoroacetate, sodium iodide, and sodium trifluoroacetate) were weighted separately and dissolved in tetrahydrofuran to prepare a 5 mg/ml solution. Two solid particle polymers (PEG-6000 and PVPK12) were weighted separately and dissolved in tetrahydrofuran to prepare a 10 mg/ml solution. 10 μL of two solution polymers (KPOP-5040 and DL-4000) was transferred into the tube separately with a sample gun and diluted to 1 ml with tetrahydrofuran. Ultrasound was used to assist in dissolving all of the above solutions and mixing according to polymer/matrix/cationization agent = 5/15/1 (v/v/v). The cationization reagent was fixed as silver trifluoroacetate.

#### Cationization Reagent Application Optimization

According to the method described in Effects of Matrix, the matrix, cationization reagent, and polymer were taken in the corresponding amount, dissolved, and diluted with 1 ml of tetrahydrofuran, respectively. Then, according to the best matrix for each polymer, polymer/matrix/cationization agent = 5/15/1 (v/v/v) was mixed with silver trifluoroacetate, sodium iodide, and sodium trifluoroacetate chosen as cationization reagents.

According to the best matrix and cationization reagent for each polymer, polymer/matrix = 5/15 μL (v/v) was mixed. Different amounts of cationization reagents (0, 1, 2, 4, 6, 8, and 10 μL) were set for mixing with the polymer and matrix with a fixed volume ratio.

#### Effects of the Matrix Solution Composition

According to the method described in Effects of Matrix, the matrix, cationization reagent, and polymer were taken in the corresponding amount, dissolved, and diluted with 1 ml of tetrahydrofuran, 1 ml methanol, and 1 ml acetone, respectively. Then, according to the best matrix and cationization reagent for each polymer, polymer/matrix/cationization agent = 5/15/1 (v/v/v) was mixed.

#### Matrix and Cationization Reagent Mixing Ratio Optimization

According to the method described in Effects of Matrix, the corresponding amount of matrix, cationization agent, and polymer were taken, respectively, dissolved, and diluted with 1 ml of tetrahydrofuran. Then, the volume of each polymer was fixed at 5 μL with the best matrix and cationization reagent. According to the selected optimal cationization reagent amount, different amounts of the matrix solution (1, 5, 15, 25, 35, 50, and 100 μL) were set for mixing.

#### Laser Intensity Optimization

The optimized matrix, cationization reagents, and their ratio of each polymer selected according to the previous experiment were applied for sample preparation; different laser energies for MALDI-TOF MS analysis are used to screen out the optimal laser energy conditions.

#### MALDI-TOF MS-Based Sample Analytical Procedure

MALDI-TOF/TOF MS analysis was performed with the SCIEX ® 5800 system. To reduce the operational error and systematic error, enzymatically hydrolyzed peptide of myoglobin from the horse was used for each sample group before analysis, as an external sample for external instrument calibration. The MS parameter was set as follows. An Nd: YAG laser at 335 nm was used with a pulse frequency of 40 Hz and acceleration voltage was set as 20 kV. The reflected positive ion mode was selected and the data are acquired as the automatic acquisition mode. The best scanning mass-to-charge ratio (m/z) range for MALDI-TOF MS was 600–12,000. Each scan accumulated 500 mass spectrum signals, and each sample accumulated 2,000 signals. Peak identifications were performed using Data Explorer software (version 4.5).

## Results and Discussion

### Effect of Matrix on Polymer Ionization Efficiency

**FIGURE 1:**
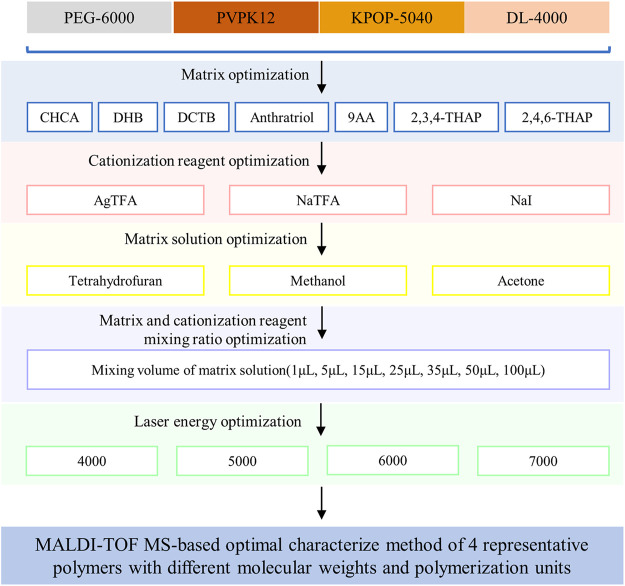
Experiment scheme. Workflow of the study.

As a small molecule compound, the matrix has the function of absorbing laser energy and transferring protons in the MALDI process. Generally, the compatibility of the matrix and the polymer is the first thing to be considered when choosing the matrix for polymer analysis (Dwyer, 1996). Only by choosing a matrix that is relatively close to the hydrophilicity/hydrophobicity of the polymer can the uniformity of the two mixtures be ensured, including the uniformity of the solution and the uniformity of the crystals on the target after cocrystallization (Tummala and Limbach, 2010). In this study, MALDI-TOF MS was used to characterize four representative polymers including PEG-6000, PVPK12, KPOP-5040, and DL-4000. The preparation methods of these four polymer samples have been optimized from six aspects, including matrix, cationization reagent, solvent, mixing ratio of cationization reagent to polymer, mixing ratio of matrix to polymer, and laser intensity (Figure 1).

**FIGURE 2:**
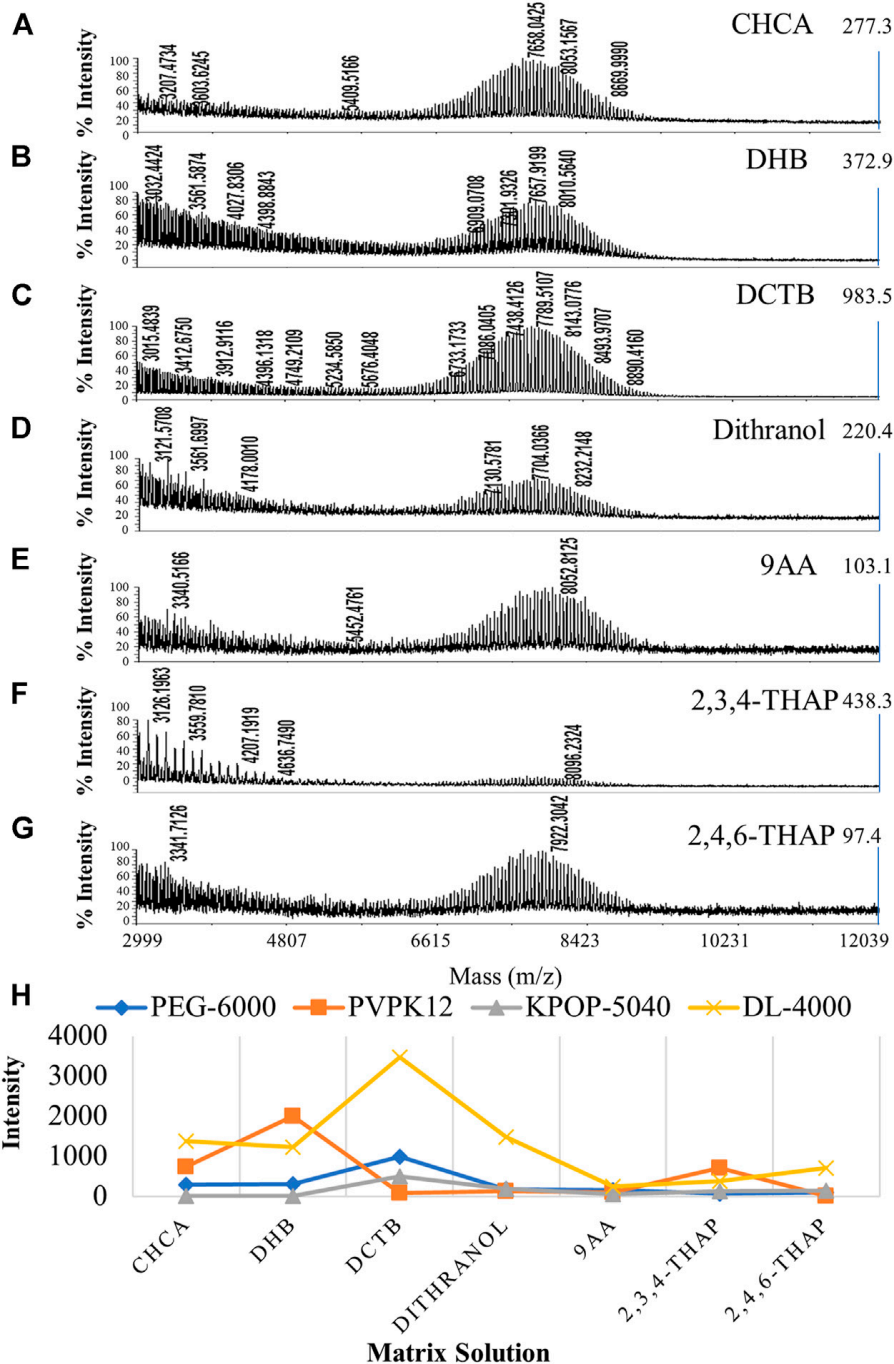
MALDI-TOF MS spectrums of PEG-6000 with different matrices including CHCA **(A)**, DHB **(B)**, DCTB **(C)**, dithranol **(D)**, 9AA **(E)**, 2,3,4-THAP **(F),** and 2,4,6-THAP **(G)**. Effect of the matrix on polymer ionization efficiency **(H)**. Blue, orange, gray, and yellow colors indicate the peak intensity of PEG-6000, PVPK12, KPOP-5040, and DL-4000, respectively.

Firstly, we mixed the fixed volume ratio of polymer, matrix, and cationization reagent to 5/15/1(v/v/v) based on experience. Silver trifluoroacetate was used as a fixed cationization reagent for all polymers, in order to examine the effects of different matrices on the ionization efficiency of the four polymers separately. Figure 2 shows the results of PEG-6000 detection on seven matrices, including CHCA, DHB, DCTB, dithranol, 9AA, 2,3,4-THAP, and 2,4,6-THAP (Figures 2A–G). It was suggested that different polymers have preferences for different matrices (Figure 2H). In our experiments, DCTB, DHB, dithranol, and CHCA were considered to have better ionization effects than the other three matrices. Interestingly, DCTB was the only matrix that had good effects for all four polymers. This result suggested that DCTB was a good matrix for polymers with different structures or molecular weights. It was shown that 2,3,4-THAP and 2,4,6-THAP as the matrix have the most unsatisfactory results, indicating that these two matrices with similar structures were not suitable for analysis. Since the use of DHB required a stronger laser, a large number of fragment ion peaks were generated in the low molecular weight region. However, DHB required a higher energy laser, and the uniformity of the crystal was not as effective as DCTB.

### Effect of Cationization Reagent on Polymer Ionization Efficiency

Analytes with strong proton affinity (containing amino groups) were easily ionized by most matrices. However, for polymers, it is difficult for conventional acidic matrices to provide a suitable ionization strategy (Biemann, 1994; Otto et al., 2010). For these special analytes, it is very necessary to choose a suitable cationization reagent (Brandt et al., 2010). The correct choice of the cationization reagent depends on the chemical properties of the analyte. The addition of alkaline ions is considered to be the best way to help polymers without containing amino groups to be ionized. Considering that most polymers were dissolved in organic solvents with higher volatility before MALDI-TOF MS analysis, basic salts with better compatibility with organic solvents, such as trifluoroacetate (Evason et al., 2000), are selected. It is valuable to consider the affinities of different polymers with basic ions, especially those polymers whose functional group charge properties differ from those of basic ions.

**FIGURE 3:**
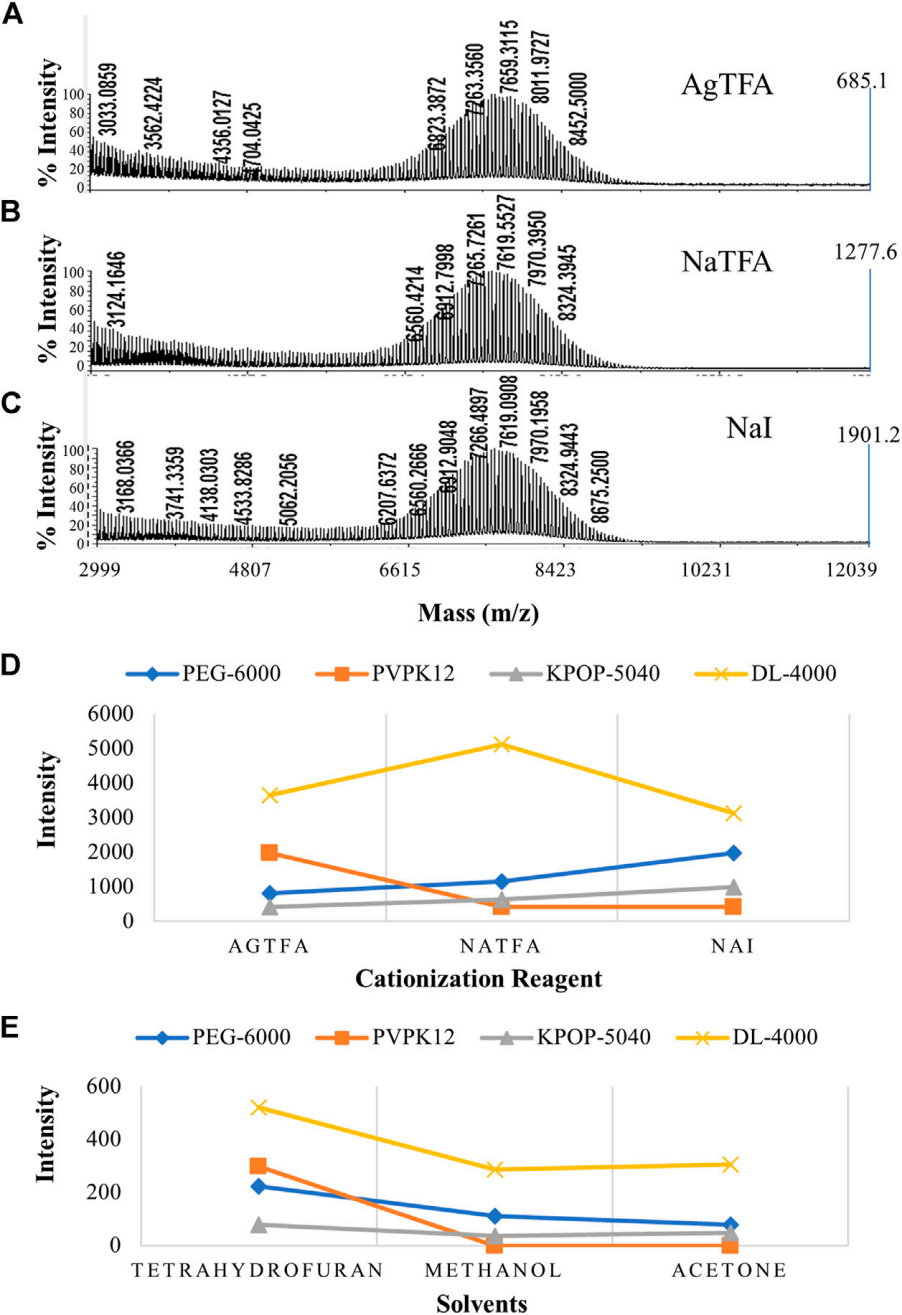
MALDI-TOF MS spectrums of PEG-6000 with different cationization reagents including AgTFA **(A)**, NaTFA **(B)**, and NaI **(C)**. Intensities of four polymers with different cationization reagents **(D)**. Effect of solvent on the polymer ionization efficiency **(E)**. Blue, orange, gray, and yellow colors indicate the peak intensity of PEG-6000, PVPK12, KPOP-5040, and DL-4000, respectively.

Among the four polymers selected in this experiment, except PVPK12 which selected DHB as the matrix, DCTB was selected as the matrix of the other three polymers. Then, according to the best matrix for each polymer, polymer/matrix/cationization agent = 5/15/1 (v/v/v) was mixed. The silver trifluoroacetate, sodium iodide, and sodium trifluoroacetate were the cationization reagents we chose. We explored the effects of three different cationization reagents on the ionization effect of PEG-6000 (Figures 3A–C) and recommended sodium iodide as the more appropriate one. Our research has found that different polymers have a preference for cationization reagents (Figure 3D). For example, the KPOP-5040 was suitable for sodium iodide as the cationization reagent, while AgTFA was more suitable for PVPK12. In addition, the DL-4000 prefers NaTFA.

### Effect of Solvents on Polymer Ionization Efficiency

For MALDI mass spectrometry analysis based on solution drying, the first consideration for sample preparation is to completely dissolve the analyte, matrix, and cationization reagent (Marie et al., 2000). The solubility of different analytes themselves, as well as their solubility with the matrix and cationization reagents, should be taken into consideration at the same time. The ideal situation is to choose only one solvent during the entire experiment to avoid precipitation that might be caused by multiple solvents not evaporating at the same time. In addition, to avoid matrix recrystallization over the solvent evaporation process, it is better to choose a solvent that evaporates very quickly (Schwarzinger et al., 2012). Thus, solvents with a wide range of dissolution and rapid evaporation were ideal for polymer sample preparation.

In this study, three common solvents (methanol, tetrahydrofuran, and acetone) with a wide range of dissolution and rapid evaporation were selected for comparison and optimization. Our experimental results suggested that only tetrahydrofuran had the ability to be compatible with all nine substances, in the process of dissolving four polymers, two substrates, and three cationization reagents. DCTB and PEG-6000 showed relatively poor solubility in methanol, while PEG-6000 and PVPK12 exhibited poor solubility in acetone. PEG-6000 dissolved in tetrahydrofuran showed the best ionization efficiency (Supplementary Figures S1A–C). We deduced that the most likely reason was the different polarities of the three solvents. The polarity of these three solvents is methanol > acetone > tetrahydrofuran in descending order. Since PEG-6000 is a nonpolar polymer, it has better compatibility with tetrahydrofuran, which is relatively smaller polarity. The experimental result indicated that, when tetrahydrofuran was used as the solvent, the MS spectra of the four polymers had the best quality (Figure 3E). At the same time, no obvious signal was detected when PVPK12 was dissolved in methanol and acetone, which was consistent with the poor solubility of PVPK12 over the sample dissolution process. It can be proved that the successful screening of a solvent which simultaneously dissolves the polymer, matrix, and cationization agent is essential and a success factor for the MALDI-TOF MS analysis of molecular polymers.

### Effect of Different Mixing Ratios on the Polymer Ionization Efficiency

After selecting the matrix, cationization agent, and solvent corresponding to each polymer, the mixing ratio among them also had a great effect on the ionization efficiency of the polymer. First of all, the sample, matrix, and cationization reagent should be dissolved in a reasonable concentration. When the mixture was volatilizing, precipitation would occur if the sample was the earliest one that reached the dissolution limit, and the effect of forming cocrystal crystals would be poor (Schriemer and Liang, 1997).

**FIGURE 4:**
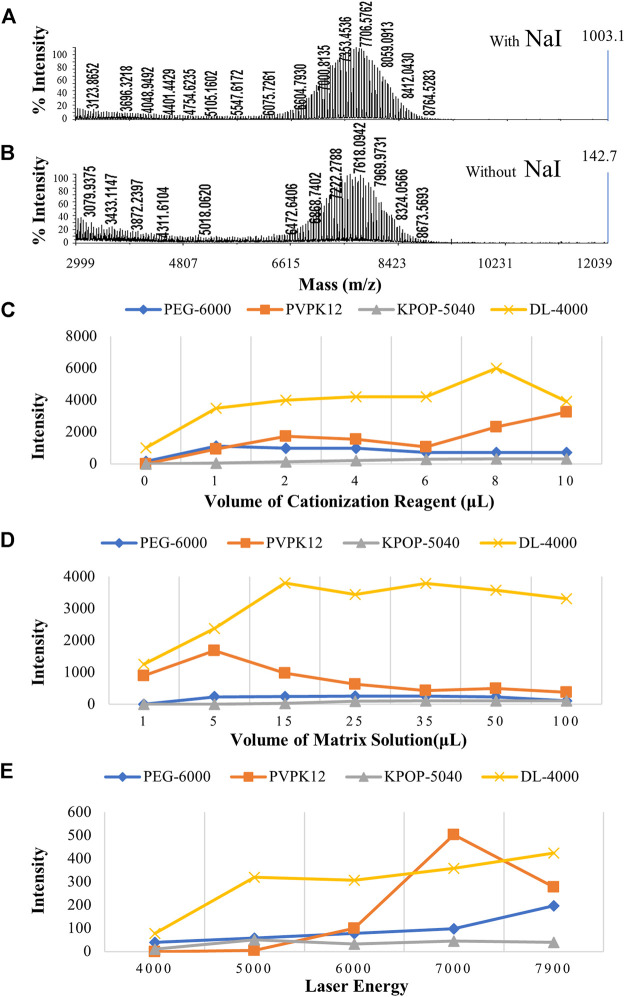
MALDI-TOF MS spectrums of polymers with the cationization reagent **(A)** and without the cationization reagent **(B)**. The effect of different ratios of the cationization reagent **(C)**, different ratios of the matrix **(D)**, and laser energy **(E)** on the polymer ionization efficiency. Blue, orange, gray, and yellow colors indicate the peak intensity of PEG-6000, PVPK12, KPOP-5040, and DL-4000, respectively.

The first step is to take into account the mixing ratio between the cationization reagent and the polymer. Under the condition of a fixed amount of matrix, the polymer peak was very weak, when the amount of cationization reagent is 0 μL, which suggested that the cationization reagent was really able to increase the intensity of the polymer signal (Figures 4A,B). However, when the amount of cationization reagent was gradually increased from 1 to 10 μL, there was no obvious difference in the effect of the four polymers, which suggested that excessive cationization reagent had no obvious effect on the improvement of the polymer signal (Figure 4C). Interestingly, the cationization reagent was found to not only increase the signal strength of the polymer, but also interfere with the analysis of MALDI-TOF MS. For example, in the analysis of PVPK12, with the increase of AgTFA concentration, the silver-added cluster peaks formed by the polymer became more and more obvious.

The second step is to take into account the mixing ratio between the matrix and the polymer. Since the selection of a suitable matrix plays a very important role in polymer analysis, a suitable mixing ratio of the matrix and polymer also has a great effect on the analysis signal (Macha et al., 2001). Generally, the matrix/polymer whose molar ratio was in the range of 100/1 to 100,000/1 was able to get good detection results (Figure 4D). However, when the polymer itself could not be ionized, such as when the amount of matrix was quite low, the peaking effect would become very poor. In this experiment, the signal strength of the four polymers was very weak when the molar ratio of the matrix to polymer was at a low level (the matrix volume was set as 1 and 5 μL). On the contrary, while the molar ratio of the matrix to the polymer was gradually increased (the matrix volume was set from 15 to 100 μL), the four polymers could obtain sufficiently strong signal intensities.

### Effect of Laser Energy on the Polymer Ionization Efficiency

For MALDI-TOF MS, in addition to the sample preparation that determines whether the analyte can be ionized under the optimal conditions, the laser energy also affects the peaking of the analyte (Cohen and Chait, 1996). In the process of MALDI source ionization, the laser plays two important roles, including providing energy for the matrix and analyte and ionizing the matrix to generate ion current (Wang et al., 1996). The sample cannot be ionized at low laser energy. Properly increasing the laser energy can significantly increase the signal of the analyte (Montaudo et al., 1995). The prerequisite is that the laser energy must be higher than the minimum energy required for sample ionization. In this study, the signals of the four polymers have been observed to be significantly improved when the laser energy was gradually increased from 4000 to about 7000 (Figure 4E and Supplementary Figures 2A–D). However, when the laser energy reached 7900, the signal of the polymer was not significantly improved as wished, but the resolution of the peak was reduced. This result might suggest that, when the laser energy was too high, it would produce negative effects. This would affect the analysis of the polymer structure from two aspects, including the reduction of resolution due to the saturation of the detector signal and fragments formed due to the fracture of the polymer.

### DSPE-PEG-NH_2_ Identification

**FIGURE 5:**
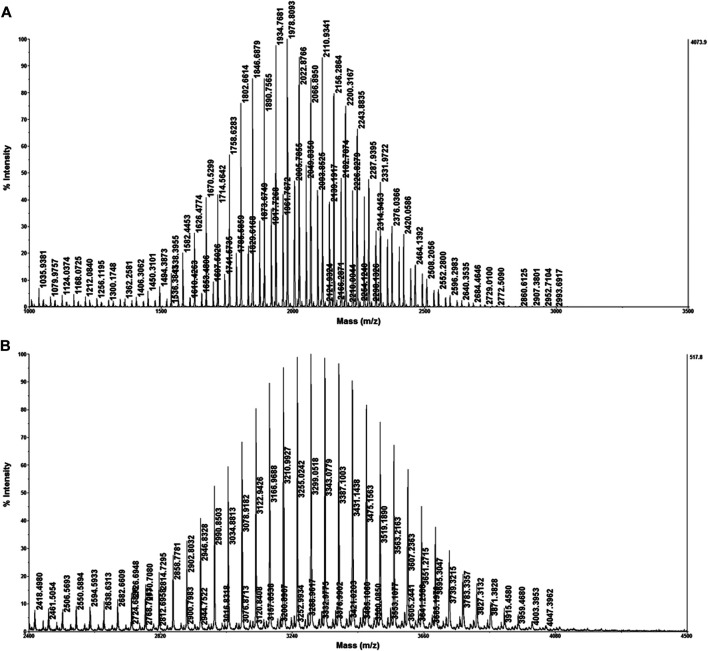
Identification of DSPE-PEG-NH2-2000 **(A)** and DSPE-PEG-NH2-3400 **(B)** with the optimized strategy.

As a significant linker for liposomal nanocarrier, different degrees of polymerized DSPE-PEG-NH_2_ were widely used in biomedical and visible probing (Kang and Ko, 2019; Rodrigues et al., 2019). We further applied the optimized method to identify DSPE-PEG-NH_2_ with different polymerized degrees. DSPE-PEG-NH_2_-2000 and DSPE-PEG-NH_2_-3400 were first dissolved in tetrahydrofuran in order to get better ionization efficiency. DCTB was chosen as the matrix due to the better compatibility for polymers. Sodium iodide was employed as the cationization reagent to achieve better ionization efficiency. As shown in Figure 5, both DSPE-PEG-NH_2_-2000 and DSPE-PEG-NH_2_-3400 were identified with excellent intensity even when the concentration was only 0.5 mg/ml.

## Conclusion

In this study, MALDI-TOF MS was used to characterize four representative polymers including PEG-6000, PVPK12, KPOP-5040, and DL-4000. The preparation methods of these four polymer samples have been optimized from six aspects, including matrix, cationization reagent, solvent, mixing ratio of cationization reagent to polymer, mixing ratio of matrix to polymer, and laser intensity. After investigating the effects of seven commonly used matrices on the ionization efficiency of four polymers, DCTB was found to be the only matrix suitable for the analysis of all the four polymers. For PVPK12, DHB was also a suitable matrix, which was helpful for improving sensitivity. Our experimental results suggested that sufficient strong signal intensities were obtained only when the molar ratio of matrix to polymer was at a certain high level. Otherwise, the effect of forming cocrystal crystals would be poor while the molar ratio of matrix to polymer was at a low level. The polymer peak was very weak without the cationization reagent. We found that it was really able to increase the intensity of the polymer signal when the amount of cationization reagent was gradually increased. However, different polymers showed a certain preference for different cationization reagents. For example, the KPOP-5040 was suitable for sodium iodide as the cationization reagent, while PVPK12 was more suitable for AgTFA. In addition, the DL-4000 prefers NaTFA. For the choice of solvent, tetrahydrofuran is a reagent with rapid evaporation and a wide range of dissolution which can achieve the best results for the analysis of four polymers. Finally, the optimization of laser energy was also crucial. The appropriate laser energy we chose was able to achieve the best ionization effect without breaking the polymer. The optimized method was successfully applied to the identification of DSPE-PEG-NH_2_ with different polymerized degrees. We have systematically analyzed the relevant factors for the high molecular polymer MALDI-MS assay and thus optimized the MALDI-MS strategy. This work provides an effective method for the fast characterization of polymers with MALDI-MS.

## Data Availability

The raw data supporting the conclusion of this article will be made available by the authors, without undue reservation.
