# Supplementary figures and images for: Optimized MALDI-TOF MS Strategy for Characterizing Polymers
Source: Front Chem. 2021 Jun 24;9:698297. doi: 10.3389/fchem.2021.698297 (PMC8264446; doi:10.3389/fchem.2021.698297)

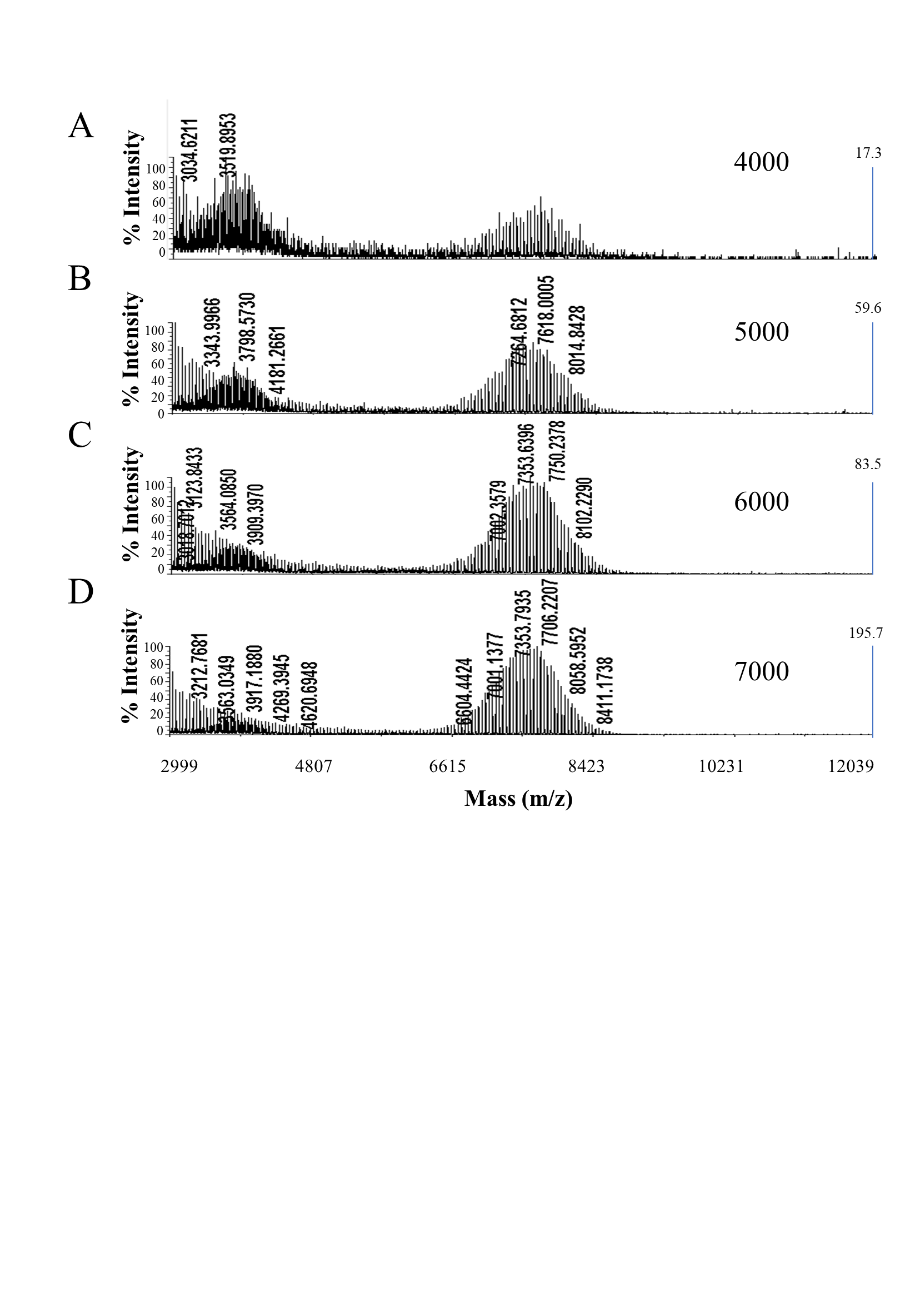

Supplement: Supplementary file 1 [file Image2.TIF]

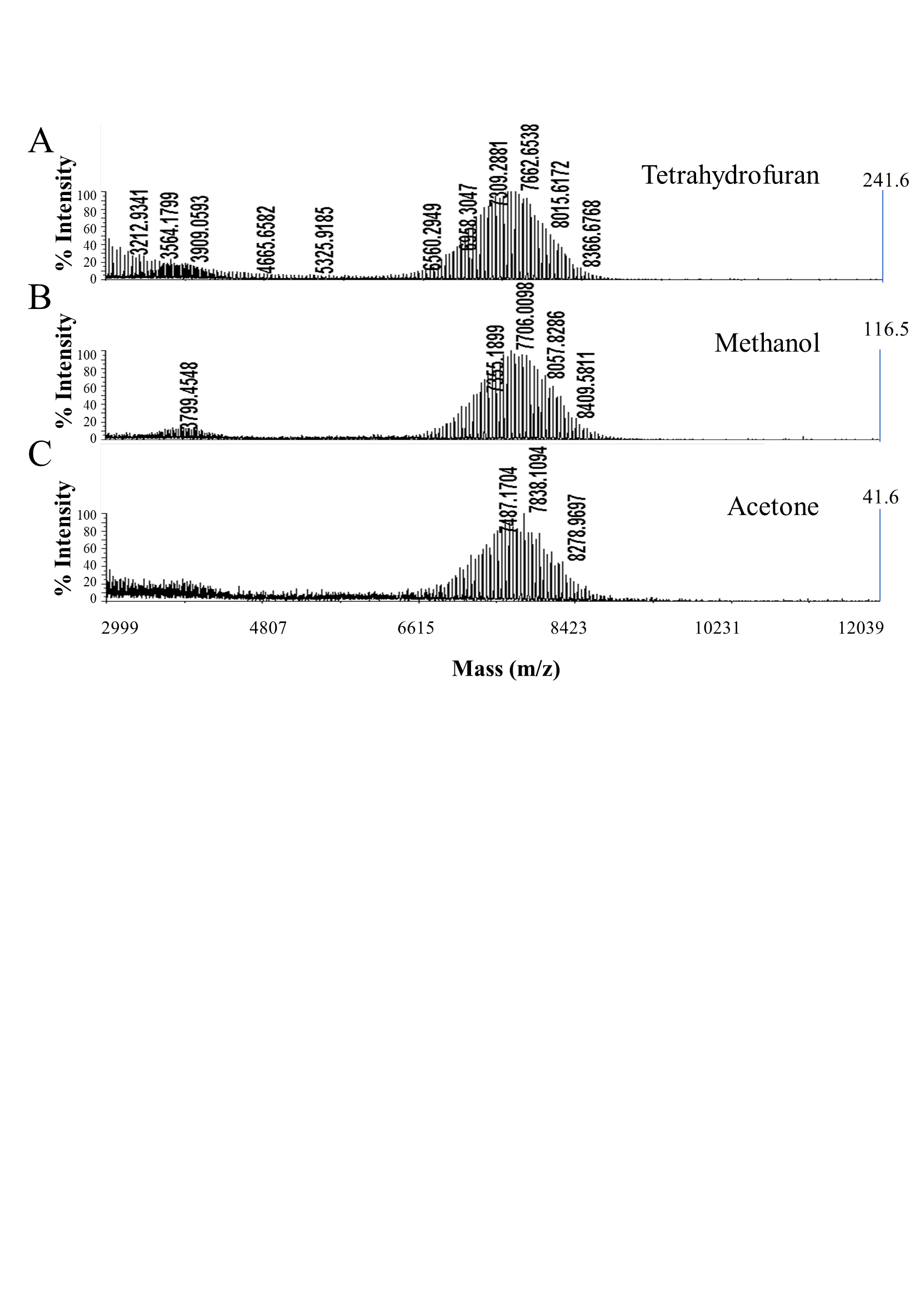

Supplement: Supplementary file 2 [file Image1.TIF]
